# Gender-Role Portrayals in Television Advertising Across the Globe

**DOI:** 10.1007/s11199-016-0617-y

**Published:** 2016-04-15

**Authors:** Jörg Matthes, Michael Prieler, Karoline Adam

**Affiliations:** 1 Department of Communication, University of Vienna, Währinger Straße 29, 1090 Vienna, Austria; 2 School of Media and Communication, Hallym University, Dasan Hall #507, Hallym University Road 1, Chuncheon, 24252 South Korea

**Keywords:** Sex role stereotyping, Social equality, Television advertising, Cross cultural differences

## Abstract

Although there are numerous studies on gender-role portrayals in television advertising, comparative designs are clearly lacking. With content analytical data from a total of 13 Asian, American, and European countries, we study the stereotypical depiction of men and women in television advertisements. Our sample consists of 1755 ads collected in May 2014. Analyzing the gender of the primary character and voiceover, as well as the age, associated product categories, home- or work setting, and the working role of the primary character, we concluded that gender stereotypes in TV advertising can be found around the world. A multilevel model further showed that gender stereotypes were independent of a country’s gender indices, including Hofstede’s Masculinity Index, GLOBE’s Gender Egalitarianism Index, the Gender-related Development Index, the Gender Inequality Index, and the Global Gender Gap Index. These findings suggest that gender stereotyping in television advertising does not depend on the gender equality prevalent in a country. The role of a specific culture in shaping gender stereotypes in television advertising is thus smaller than commonly thought.

The past four decades have witnessed a plethora of studies on gender-role portrayals in advertising on television (Cheng 1997; Furnham and Voli 1989; McArthur and Resko 1975; Milner and Collins 2000; Pacilli et al. 2016; Paek et al. 2011). From the perspective of marketers and advertisers, gender is a primary segmentation variable in developing marketing strategies and defining target groups (An and Kim 2007; Milner and Collins 2000). Also, gender portrayals have possible effects on corporate images and on purchase intentions of consumers (Ford and LaTour 1996). From the perspective of activists, advertising councils, or policy regulators, however, the stereotypical depiction of men and women in today’s ads is problematic for many reasons (Coltrane and Messineo 2000). It is argued that advertisers create and perpetuate gender stereotypes, which may erode gender equality and harm society at large (MacKay and Covell 1997; Oppliger 2007).

The accumulated empirical evidence clearly suggests that gender roles are highly stereotypical in television ads (Eisend 2010; Furnham and Mak 1999; Furnham and Paltzer 2010). However, despite this strong research tradition around the world, it is less clear how a country’s culture shapes gender roles in TV ads. This research deficit can be traced back to the research designs used in previous research. In fact, most research on gender stereotypes in television advertising is based on single-country studies (Das 2011; Kim and Lowry 2005; Uray and Burnaz 2003). Because such studies work with a specific sample at a specific time of the year, we cannot use them to compare findings across countries. For instance, if we see a difference between two studies from two different countries, we may not be sure whether the observed dissimilarities reveal cultural differences or whether they can be traced back to differences in time frame, sampling, codebook, or other characteristics of an individual study (Matthes et al. 2012). Comparative research is therefore urgently needed.

Only a very few studies have analyzed gender roles in a comparative fashion allowing for a test of country and cultural differences (An and Kim 2007; Gilly 1988; Milner and Collins 2000; Paek et al. 2011). Most of these studies have relied on one or two comparative gender indices (such as Hofstede’s), and more recent indices have not been employed. This limitation makes it difficult to come to a definite conclusion about the role of cultural variables in explaining the portrayal of men and women in TV advertising. Even more importantly, almost all the relevant studies have looked at television ads in only two or three countries (Gilly 1988; Milner and Collins 2000), which is a clear limitation in terms of detecting cultural patterns. In fact, only Paek et al. (2011) looked at television advertisements across seven countries and used Hofstede’s masculinity dimension and the gender development index to explain gender-role portrayals.

Although Paek et al.’s (2011, p. 204) study is pioneering in many aspects, they only tried to predict the gender of the primary character and the voiceover and sampled the material from different months (i.e., July vs. November), “which may reduce the generalizability and the comparability of [their] findings within and across the countries.” Also, their data are from 2002, and it remains unclear―as with most content analytical research―how the findings can be generalized to more recent years. Even more importantly, all the known prior comparative studies have not empirically modeled the influence of culture on gender stereotypes. It is not the same to interpret differences between countries by drawing on cultural differences as it is to empirically measure and confirm the role of culture in an adequate statistical model. If a specific culture shapes gender stereotypes, then a country’s score on a cultural dimension should help to explain the degree of gender stereotyping. Unfortunately, such a multilevel analysis (see Raudenbush and Bryk 2002) is not known to have been conducted in extant research.

Therefore, the aims of our study were to observe gender-role portrayals around the world, using comparable measures, sampling strategies, and classic as well as recent gender indices to explain country differences. We analyzed a total of 1755 television advertisements from 13 countries, which were selected based on their scores on various gender indices: Austria, Brazil, China, France, Germany, Japan, Netherlands, Romania, Slovakia, South Korea, Spain, United Kingdom, and United States. To date, our study is the largest known study on gender portrayals ever conducted, and we are the first to employ the concept of gender egalitarianism from Project GLOBE (House et al. 2004) and various other gender indices at the same time.

## Gender-Role Portrayals in Advertising

Research on gender role portrayals in U.S. television advertisements started in the 1970s (Dominick and Rauch 1972; McArthur and Resko 1975), followed by research in Great Britain and Europe in the 1980s (Furnham and Voli 1989; Manstead and McCulloch 1981) and in Asia in the 1990s (Cheng 1997; Sengupta 1995). Generally, this research has led to the consensus that gender roles are highly stereotypical in television advertisements (Eisend 2010; Furnham and Paltzer 2010) across a range of commonly explored variables. The most frequently analyzed variables are the gender of the primary character, the gender of the voiceover, the age of the primary character, and the setting associated with the primary character.

Analyses of the gender of the primary character led to rather mixed results in previous research, with some studies showing male predominance, others showing a female predominance, and still others finding almost no difference. Nevertheless, the majority of studies showed a predominance of male primary characters (see Eisend 2010; Furnham and Paltzer 2010). Some research has indicated a relation between Hofstede’s masculinity index and the gender predominance of the primary character (Milner and Collins 2000), whereas others found the predictive role of Hofstede’s masculinity index to be minimal (Paek et al. 2011). We thus hypothesize that there will be more men than women depicted as primary characters in our analyses (Hypothesis 1).

The age of the primary character is another widely studied variable. Most studies report a predominance of women in the younger age segment (under 35), whereas more men were found in the middle and older age segments (Furnham and Mak 1999; Furnham and Paltzer 2010). In a meta-analysis, Eisend (2010) found that the odds of women being younger are three times higher than for men. Hence, there are strong grounds to expect that female primary characters will be depicted as younger compared to male characters (Hypothesis 2).

Furthermore, the predominance of male voiceovers (which was often interpreted as the “voice of authority”) is one of the most consistent findings in the literature—a predominance that is even more pronounced in Asia compared to other regions (Furnham and Mak 1999). Previous research has indicated that a higher score in Hofstede’s masculinity index increases the odds of a male voiceover (Paek et al. 2011). Thus, we predict that there will be more male voiceovers compared to female voiceovers in television ads (Hypothesis 3).

In terms of product categories associated with a specific gender, there are relatively few consistent findings; this may be because different studies often employ different product categories. One finding that was confirmed in most studies, however, was the association between women and body products, or as other studies called them, “toiletries,” “beauty products,” and “personal care products,” as well as “household and cleaning products” (Furnham and Paltzer 2010). For men, product associations were less clear, but some studies found associations between men and television advertisements for cars, telecommunications, electronics, technology, and computers (Ganahl et al. 2003; Royo-Vela et al. 2008). Based on these findings, we expect that female primary characters will be more likely to be seen in ads for toiletries, beauty products, and cleaning products (Hypothesis 4a), whereas male characters will be associated more with telecommunications, electronics, technology, computers, or cars (Hypothesis 4b).

The setting is another variable that has often produced clear gender divisions. Most often cited is the association of women with a home setting (Das 2011; Uray and Burnaz 2003; Valls-Fernández and Martínez-Vicente 2007). A meta-analysis shows that the odds of women being depicted at home (vs. at work) are approximately 3.5 times higher than for men (Eisend 2010). Another finding that was similar across most of the literature is that more men than women are shown in a workplace setting (Prieler and Centeno 2013; Valls-Fernández and Martínez-Vicente 2007). Therefore, we hypothesize that female primary characters will be more likely to be depicted in a home setting (Hypothesis 5a), whereas male characters will be more likely to be shown at work (Hypothesis 5b).

In addition to these commonly analyzed variables, we focused on the working role of the primary character (no working role, high status worker, and lower status worker). Although categorizations differed, several studies investigated whether the primary character was working (Coltrane and Messineo 2000; Das 2011; Uray and Burnaz 2003) or was a homemaker (Uray and Burnaz 2003; Valls-Fernández and Martínez-Vicente 2007)—both leading to highly stereotypical results that suggested more men than women are working and more women than men are depicted as homemakers. Thus we expect female primary characters will be more likely to be seen in lower status working roles compared to male primary characters, who will be more likely to be seen in higher status roles (Hypothesis 6).

## Cultural Models, Gender Indices, and Advertising

To fully understand gender-role portrayals, scholars have repeatedly pointed to the important role of cultural differences. The most widely applied cultural model in advertising research is Hofstede’s cultural dimensions (Okazaki and Mueller 2007), including four dimensions (he later added two additional ones): power distance, individualism/collectivism, uncertainty avoidance, and the masculinity dimension (De Mooji and Hofstede 2010; Hofstede 2001). The most relevant dimension for the purposes of the present paper is Hofstede’s masculinity dimension, which has been used in cross-cultural content analyses on gender in advertising, including studies comparing two countries (An and Kim 2007; Huang 1995; Moon and Chan 2002; Odekerken-Schröder et al. 2002) and some comparing three or more countries (Milner 2005; Milner and Collins 1998, 2000; Paek et al. 2011; Wiles et al. 1995). However, only a few studies have confirmed an association between the masculinity index and gender portrayals (Huang 1995; Wiles et al. 1995), whereas more studies have led to mixed results (An and Kim 2007; Milner and Collins 2000) or to results that were mostly opposite from those predicted by Hofstede’s masculinity index (Milner 2005; Moon and Chan 2002; Odekerken-Schröder et al. 2002; Paek et al. 2011).

Considering the mixed results of previous research in general and criticisms of Hofstede’s (2001) study in particular—as being rather outdated (An and Kim 2007; Okazaki and Mueller 2007) and for its masculinity dimension that mixes two sub-dimensions (i.e., the characteristics of a society and the gender role distinctions) (Emrich et al. 2004; Hofstede 2001)—it is crucial to use additional indices, such as the more recent framework from the GLOBE project (House et al. 2004). Its theoretical importance and promise have been mentioned in several articles on theory in advertising research (Okazaki and Mueller 2007; Taylor 2010). Still, the GLOBE study has been used rarely in gender stereotyping research to date. In contrast to Hofstede’s study, the GLOBE project differentiates between societal practices and values. Practices are measured through questions regarding “what is,” while values are measured through questions regarding “what should be” (House et al. 2010).

We have decided to use societal practices in our study because gender portrayals are about the way a society actually performs, whereas values are about how a society should perform (House et al. 2010; Okazaki et al. 2010). Although Hofstede’s (2001) masculinity index influenced the GLOBE project (the GLOBE dimensions include: Performance Orientation, Uncertainty Avoidance, Human Orientation, Institutional Collectivism, In-Group Collectivism, Assertiveness, Gender Egalitarianism, Future Orientation, and Power Distance), the latter separated this dimension into gender egalitarianism and assertiveness. *Gender egalitarianism* was defined as “the degree to which a society minimizes gender role differences while promoting gender equality” (House et al. 2010, p. 118). Gender egalitarianism reflects society’s beliefs about whether biological sex should determine roles in society; gender egalitarian societies rely less on biological sex to make those determinations (Emrich et al. 2004). In such societies, there is less occupational sex segregation, more women in the labor force and in positions of authority, and generally a higher status for women. Thus, gender egalitarianism is clearly related to our study.

Despite the call to use Project GLOBE’s dimensions in advertising research (House et al. 2010; Okazaki and Mueller 2007), only a few studies to date have employed them. These studies have included the dimensions of assertiveness (Okazaki et al. 2010; Terlutter et al. 2010), performance orientation (Okazaki et al. 2010), and humane orientation (Diehl et al. 2012), and they have indicated an association between GLOBE dimensions and advertising evaluations. Thus, no known study to date has used gender egalitarianism as a theoretical framework or used Project GLOBE dimensions in content analysis.

In addition to testing the predictive power of cultural models for gender stereotyping, we take into account other dimensions related to gender development because previous research has indicated that gender role portrayals may be influenced by a country’s gender development (Eisend 2010; Paek et al. 2011). Paek et al. (2011) found that the predictive role of the Gender-related Development Index (GDI) by the United Nations Development Program (UNDP) seems to be minimal for the gender of the prominent character in an advertisement. However, they also reported that the odds of using a male voiceover significantly increased as GDI scores dropped. In his meta-analysis, Eisend (2010) used another gender index created by the UNDP—namely, the Gender Empowerment Measure (GEM)—and found a correlation between the GEM and gender stereotyping in advertising.

Our research extends these studies by testing whether the GDI (UNDP 2014a), the UNDP’s Gender Inequality Index (GII) (UNDP 2014b), which replaced the GEM due to criticism, and the World Economic Forum’s Gender Gap Index (GGGI) can predict gender stereotypes (Hausmann et al. 2014). All of these indices are based on demographic data. The GDI is based on gender gaps in life expectancy, education, and incomes; the GII on reproductive health, empowerment (such as share of parliamentary seats and higher education levels) and labor market participation; and the GGGI is the most inclusive, being based on gender gaps on economic, political, educational, and health criteria (Hausmann et al. 2014; UNDP 2014a, b). Furthermore, besides Hofstede (2001), our study uses GLOBE for the first time in the extant research on gender portrayals as a theoretical framework (House et al. 2004). Because all indices differ and previous evidence of their impact on gender portrayals is mixed at best, we ask as an exploratory research question how the five indices (Hofstede’s masculinity index, the Project GLOBE’s gender egalitarianism index, the GDI, the GII, and the GGGI) predict gender-role portrayals in television advertising.

## Method

We analyzed gender role stereotypes across Asia, America, and Europe (Austria, Brazil, China, France, Germany, Japan, Netherlands, Romania, Slovakia, South Korea, Spain, United Kingdom, and United States). We selected the countries based on the following criteria. First, we wanted to have a broad range of countries that have different scores on various gender indices. For that reason, we have included countries with a high score on Hofstede’s masculinity index (Japan, Slovakia, and Austria) and with a low score (Netherlands), as well as countries with a high score on GLOBE’s gender egalitarianism index (United Kingdom, Netherlands, and France) and a low score (South Korea). Second, we wanted to include the countries that were frequently sampled in previous studies, for instance, the United Kingdom and the United States. Finally, the selection was driven by practical reasons, such as access to TV channels and the language qualifications of our students/coders. The most recent data from all gender indices were used.

In May 2014, 15 h of prime-time TV programming were recorded from those broadcasters with the largest shares of viewers for each respective country. We focused on private channels because we were not interested in the effects of country-specific regulations of public-service broadcasters. For Austria, we took the largest private channel as well as the largest public service channel because the audience share of the private channel is rather low. For China, we took CCTV-1 as the clearly dominating TV channel.

The recording time was split into three typical weekdays of the same week, that is, 3 × 5 h per channel. The sample included weekdays and weekends to ensure a higher diversity of advertisements (May 14/Wednesday, May 16/Friday, and May 18/Sunday). We started the recordings after Mother’s Day in most of the recorded countries to ensure that no large country- and/or region-specific events occurred during the days of recording. We focused on prime time because it was commonly used in previous studies (Paek et al. 2011). Because the definition of prime time varied by country, we used the most inclusive definition (i.e., from 6 PM to 11 PM). Like in most prior research (see Kim and Lowry 2005), duplicate ads, political ads, ads for films and CD’s, public service announcements (PSAs), and ads with children, animals, or comic figures as dominant actors were not considered in our study. After 150 ads were collected in one country, we stopped additional data collection; however, the 3 days sampled produced less than 150 unique advertisements for several countries. Our sample consisted of 1755 ads. These included ads from Austria (ORF2, *n* = 98; Puls4, *n* = 124), Germany (RTL, *n* = 144), the United Kingdom (ITV1, *n* = 149), the United States (ABC, *n* = 149), France (TF1, *n* = 150), Spain (Telecinco, *n* = 146), Brazil (Rede Globo, *n* = 123), Netherlands (RTL4, *n* = 149), Romania (ProTV, *n* = 115), Slovakia (TV Markiza, *n* = 118), China (CCTV-1, *n* = 137), Japan (NTV, *n* = 150), and South Korea (SBS, *n* = 127).

### Coding Procedure and Reliability

The codebook was adopted from prior research (Prieler and Centeno 2013; Furnham and Paltzer 2010; Nassif and Gunter 2008). The following categories were included in the analysis: primary character, product category, voiceover/narrator, age, dominant setting, and working role. These are standardized measures in this research (see Appendix Table 6 for codes). The coding team consisted of 30 coders from two major universities in South Korea and Austria. Coders were one of the authors and 29 undergraduate students receiving compensation/course credit for their work. Students were unaware of the hypotheses and were native or bilingual in the language of the country they coded. Training sessions on using the codebook were held with all coders prior to testing reliability. Ads were randomly selected for the reliability test but had to involve a primary character so that all codebook categories could be included. We performed three reliability tests using Krippendorff’s Alpha (Hayes and Krippendorff 2007; *n* = 10 each, from 2 to 30 coders). One test was between coders for different countries on English-language advertisements, one test was between coders coding the same country prior to coding, and one test used the coded material without coders knowing they were tested. Although 65 % of the reliability measures in these three tests were above *α* = .80, the lowest intercoder results for the numerous intercoder reliability tests were for primary character (*α* = .80), voiceover/narrator (*α* = .89), age (*α* = .72), dominant setting (*α* = .72), and working role (*α* = .63). This was still above the recommended chance-corrected agreement of .60 by Neuendorf (2011).

## Results

### Primary Character and Voiceover

In our first hypothesis, we stated that there will be more male than female primary characters. Among all spots with a primary character, 50.7 % of the characters were women. As can be seen in Table 1, in some countries (Brazil and Korea), the share of male primary characters was slightly higher than those of female primary characters. In other countries, however, the share was almost identical. Thus, we cannot find a substantial male predominance of primary characters. We also hypothesized (Hypothesis 2) that female primary characters would be depicted as younger compared to male primary characters. As Table 1 reveals, this pattern was the case in seven countries (Austria, Germany, France, Spain, Slovakia, Japan, USA). For the other countries, however, there was no significant effect. Based on previous research, we assumed in Hypothesis 3 that we would find more male than female voiceovers. When looking across countries (see Table 1), the share of male voiceovers (61.8 %) was, in fact, significantly higher (*p* < .01) than the share of female voiceovers (32 %). Confirming Hypothesis 3, this result was mirrored in most countries. The opposite effect, however, was found for France, with 58 % female voiceovers.

**Table 1:** Female and male primary character, age of male and female primary characters, and female and male voiceovers by country

Country	Primary character		Age				Voiceover		
	Men	Women	Men		Women		Men	Women	Both
			18–34 years	35 and older	18–34 years	35 and older			
Austria									
*n* (%)	94 (53 %)	84 (47 %)	36 (38 %)	58 (62 %)	56 (67 %)	28 (33 %)	126 (59 %)	70 (33 %)	17 (8 %)
*χ* ^2^	.56, *p* = .45		**14.30,** ***p*** **< .001**				**16.00,** ***p*** **< .001**		
Brazil									
*n* (%)	48 (65 %)	26 (35 %)	20 (42 %)	28 (58 %)	16 (62 %)	10 (39 %)	90 (83 %)	15 (14 %)	4 (4 %)
*χ* ^2^	**6.54,** ***p*** **= .01**		2.67, *p* = .10				**53.57,** ***p*** **< .001**		
China									
*n* (%)	42 (60 %)	28 (40 %)	12 (29 %)	30 (71 %)	14 (50 %)	14 (50 %)	112 (90 %)	6 (5 %)	6 (5 %)
*χ* ^2^	2.80, *p* = .09		3.30, *p* = .07				**95.22,** ***p*** **< .001**		
France									
*n* (%)	51 (46 %)	59 (54 %)	23 (45 %)	28 (55 %)	48 (81 %)	11 (19 %)	49 (36 %)	80 (58 %)	9 (7 %)
*χ* ^2^	.58, *p* = .45		**15.71,** ***p*** **< .001**				**7.45,** ***p*** **< .01**		
Germany									
*n* (%)	47 (42 %)	65 (58 %)	19 (40 %)	28 (60 %)	49 (75 %)	16 (25 %)	90 (64 %)	45 (32 %)	6 (4 %)
*χ* ^2^	2.89, *p* = .09		**13.98,** ***p*** **< .001**				**15.00,** ***p*** **< .001**		
Japan									
*n* (%)	53 (45 %)	64 (55 %)	11 (21 %)	42 (79 %)	34 (53 %)	30 (47 %)	72 (67 %)	29 (27 %)	7 (7 %)
*χ* ^2^	1.03, *p* = .31		**12.84,** ***p*** **< .001**				**18.31,** ***p*** **< .001**		
Netherlands									
*n* (%)	57 (52 %)	52 (48 %)	36 (63 %)	21 (37 %)	37 (71 %)	15 (29 %)	72 (55 %)	55 (42 %)	4 (3 %)
*χ* ^2^	.23, *p* = .63		.79, *p* = .38				2.28, *p* = .13		
Romania									
*n* (%)	35 (45 %)	43 (55 %)	24 (69 %)	11 (31 %)	37 (86 %)	6 (14 %)	85 (76 %)	23 (21 %)	4 (4 %)
*χ* ^2^	.82, *p* = .37		3.46, *p* = .06				**35.59,** ***p*** **< .001**		
Slovakia									
*n* (%)	41 (47 %)	46 (53 %)	17 (42 %)	24 (59 %)	30 (67 %)	15 (33 %)	72 (64 %)	40 (35 %)	1 (1 %)
*χ* ^2^	.29, *p* = .59		**5.50,** ***p*** **= .02**				**9.14,** ***p*** **= .002**		
South Korea									
*n* (%)	66 (64 %)	38 (37 %)	28 (42 %)	38 (58 %)	22 (58 %)	16 (42 %)	43 (39 %)	39 (36 %)	28 (26 %)
*χ* ^2^	**7.54,** ***p*** **= .01**		2.31, *p* = .13				.20, *p* = .66		
Spain									
*n* (%)	36 (42 %)	50 (58 %)	11 (31 %)	25 (69 %)	28 (56 %)	22 (44 %)	79 (60 %)	44 (33 %)	9 (7 %)
*χ* ^2^	2.28, *p* = .13		**5.47,** ***p*** **= .02**				**9.96,** ***p*** **= .002**		
UK									
*n* (%)	50 (49 %)	52 (51 %)	12 (24 %)	38 (76 %)	20 (39 %)	32 (62 %)	58 (48 %)	59 (48 %)	5 (4 %)
*χ* ^2^	.04, *p* = .84		2.48, *p* = .12				.01, *p* = .93		
USA									
*n* (%)	60 (52 %)	55 (48 %)	16 (27 %)	44 (73 %)	33 (60 %)	22 (40 %)	90 (71 %)	33 (26 %)	4 (3 %)
*χ* ^2^	.22, *p* = .64		**13.04,** ***p*** **< .001**				**26.42,** ***p*** **< .001**		

For Female and Male Primary Character and Female and Male Voiceovers, a *χ* ^2^ goodness-of-fit test was used

### Product, Setting, and Work Role

In line with prior research, we expected that female primary characters would be more likely to be seen in ads for toiletries, beauty products, personal care, and cleaning products, whereas male characters would more likely be associated with telecommunications, electronics, technology, computers, or cars. The findings are reported in Table 2. In all countries but Japan, the association of female primary characters with toiletries, beauty products, personal care, and cleaning products can be confirmed. The association of male primary characters with products related to technology and cars (see Table 2) was observed for Brazil, Germany, the Netherlands, Spain, and the United Kingdom. The effect was not significant by the conventional *p* < .05 level for Romania, Slovakia, Austria, USA, China, Japan, and South Korea.

**Table 2:** Association of body or cleaning products, technical products and cars with the primary characters by country

Country	Body or cleaning products				Technical products or cars			
	Men		Women		Men		Women	
	No	Yes	No	Yes	No	Yes	No	Yes
Austria								
*n* (%)	87 (93 %)	7 (7 %)	52 (62 %)	32 (38 %)	82 (87 %)	12 (13 %)	77 (92 %)	7 (8 %)
*χ* ^2^	**24.35,** ***p*** **< .001**				.91, *p* = .34			
Brazil								
*n* (%)	45 (94 %)	3 (6 %)	18 (69 %)	8 (31 %)	32 (67 %)	16 (33 %)	23 (89 %)	3 (12 %)
*χ* ^2^	**8.01,** ***p*** **= .005**				**4.20,** ***p*** **= .04**			
China								
*n* (%)	41 (98 %)	1 (2 %)	23 (82 %)	5 (18 %)	34 (81 %)	8 (19 %)	22 (79 %)	6 (21 %)
*χ* ^2^	**5.14,** ***p*** **= .02**				.60, *p* = .81			
France								
*n* (%)	42 (82 %)	9 (18 %)	24 (41 %)	35 (59 %)	40 (78 %)	11 (22 %)	58 (98 %)	1 (2 %)
*χ* ^2^	**19.80,** ***p*** **< .001**				**11.12,** ***p*** **= .001**			
Germany								
*n* (%)	39 (83 %)	8 (17 %)	37 (57 %)	28 (43 %)	38 (81 %)	9 (19 %)	63 (97 %)	2 (3 %)
*χ* ^2^	**8.49,** ***p*** **= .01**				**7.96,** ***p*** **= .01**			
Japan								
*n* (%)	48 (91 %)	5 (9 %)	52 (81 %)	12 (19 %)	49 (93 %)	4 (8 %)	59 (92 %)	5 (8 %)
*χ* ^2^	2.03, *p* = .16				.00, *p* = .96			
Netherlands								
*n* (%)	54 (95 %)	3 (5 %)	36 (69 %)	16 (31 %)	46 (81 %)	11 (19 %)	50 (96 %)	2 (4 %)
*χ* ^2^	**12.29,** ***p*** **< .001**				**6.18,** ***p*** **= .01**			
Romania								
*n* (%)	32 (91 %)	3 (9 %)	32 (74 %)	11 (26 %)	26 (74 %)	9 (26 %)	39 (91 %)	4 (9 %)
*χ* ^2^	3.79, *p* = .05				3.74, *p* = .05			
Slovakia								
*n* (%)	34 (83 %)	7 (17 %)	21 (46 %)	25 (54 %)	32 (78 %)	9 (22 %)	42 (91 %)	4 (9 %)
*χ* ^2^	**12.95,** ***p*** **< .001**				3.00, *p* = .08			
South Korea								
*n* (%)	63 (96 %)	3 (5 %)	31 (82 %)	7 (18 %)	51 (77 %)	15 (23 %)	33 (87 %)	5 (13 %)
*χ* ^2^	**5.34,** ***p*** **= .02**				1.42, *p* = .23			
Spain								
*n* (%)	35 (97 %)	1 (3 %)	36 (72 %)	14 (28 %)	23 (64 %)	13 (36 %)	49 (98 %)	1 (2 %)
*χ* ^2^	**9.25,** ***p*** **= .002**				**17.87,** ***p*** **< .001**			
UK								
*n* (%)	46 (92 %)	4 (8 %)	40 (77 %)	12 (23 %)	43 (86 %)	7 (14 %)	51 (98 %)	1 (2 %)
*χ* ^2^	**4.38,** ***p*** **= .04**				**5.14,** ***p*** **= .02**			
USA								
*n* (%)	59 (98 %)	1 (2 %)	44 (80 %)	11 (20 %)	43 (72 %)	17 (28 %)	43 (78 %)	12 (22 %)
*χ* ^2^	**10.32,** ***p*** **< .001**				.65, *p* = .42			

Another prominent finding in previous research is that female primary characters are more likely to be shown in a home setting, whereas male characters are more likely to be associated with a work setting. Table 3 shows the findings for each country. The stronger depiction of female primary characters in home settings compared to male primary characters can be confirmed in Brazil, China, Germany, the Netherlands, Romania, South Korea, and Spain. There was no significant effect for six countries: Austria, France, Japan, Slovakia, USA, and United Kingdom. When it comes to the dominant depiction of male primary characters in work settings (see Table 3), we observed significant associations for Austria, Germany, France, Japan, the Netherlands, and the United Kingdom, but no significant effect for South Korea, Brazil, the United States, Spain, Romania, Slovakia, and China.

**Table 3:** Association of home setting and work setting with the primary characters by country

Country	Home setting				Work setting			
	Men		Women		Men		Women	
	No	Yes	No	Yes	No	Yes	No	Yes
Austria								
*n* (%)	70 (75 %)	24 (25 %)	59 (70 %)	25 (30 %)	81 (86 %)	13 (14 %)	81 (96 %)	3 (4 %)
*χ* ^2^	.40, *p* = .53				**5.71,** ***p*** **= .02**			
Brazil								
*n* (%)	34 (71 %)	14 (29 %)	10 (39 %)	16 (61 %)	37 (77 %)	11 (23 %)	24 (92 %)	2 (8 %)
*χ* ^2^	**7.33,** ***p*** **= .01**				2.70, *p* = .10			
China								
*n* (%)	36 (86 %)	6 (14 %)	16 (57 %)	12 (43 %)	35 (83 %)	7 (17 %)	25 (89 %)	3 (11 %)
*χ* ^2^	**7.18,** ***p*** **= .01**				.49, *p* = .49			
France								
*n* (%)	39 (77 %)	12 (24 %)	39 (66 %)	20 (34 %)	39 (77 %)	12 (24 %)	56 (95 %)	3 (5 %)
*χ* ^2^	1.43, *p* = .23				**7.90,** ***p*** **= .01**			
Germany								
*n* (%)	41 (87 %)	6 (13 %)	45 (69 %)	20 (31 %)	40 (85 %)	7 (15 %)	83 (97 %)	2 (3 %)
*χ* ^2^	**4.96,** ***p*** **= .03**				**5.15,** ***p*** **= .02**			
Japan								
*n* (%)	44 (83 %)	9 (17 %)	49 (77 %)	15 (23 %)	42 (79 %)	11 (21 %)	60 (94 %)	4 (6 %)
*χ* ^2^	.74, *p* = .39				**5.46,** ***p*** **= .02**			
Netherlands								
*n* (%)	46 (81 %)	11 (19 %)	33 (64 %)	19 (37 %)	39 (68 %)	18 (32 %)	47 (90 %)	5 (10 %)
*χ* ^2^	**4.05,** ***p*** **= .04**				**7.88,** ***p*** **= .01**			
Romania								
*n* (%)	30 (86 %)	5 (14 %)	23 (54 %)	20 (47 %)	29 (83 %)	6 (17 %)	40 (93 %)	3 (7 %)
*χ* ^2^	**9.20,** ***p*** **< .001**				1.95, *p* = .16			
Slovakia								
*n* (%)	33 (81 %)	8 (20 %)	31 (67 %)	15 (33 %)	36 (88 %)	5 (12 %)	37 (80 %)	9 (20 %)
*χ* ^2^	1.91, *p* = .17				.87, *p* = .35			
South Korea								
*n* (%)	52 (79 %)	14 (21 %)	17 (45 %)	21 (55 %)	57 (86 %)	9 (14 %)	37 (97 %)	1 (3 %)
*χ* ^2^	12.52, *p* < .001				3.36, *p* = .07			
Spain								
*n* (%)	30 (83 %)	6 (17 %)	31 (62 %)	19 (38 %)	30 (83 %)	6 (17 %)	46 (92 %)	4 (8 %)
*χ* ^2^	**4.62,** ***p*** **= .03**				1.53, *p* = .22			
UK								
*n* (%)	35 (70 %)	15 (30 %)	28 (54 %)	24 (46 %)	42 (84 %)	8 (16 %)	50 (96 %)	2 (4 %)
*χ* ^2^	2.22, *p* = .09				**4.26,** ***p*** **= .04**			
USA								
*n* (%)	50 (83 %)	10 (17 %)	43 (78 %)	12 (22 %)	48 (80 %)	12 (20 %)	48 (87 %)	7 (13 %)
*χ* ^2^	.49, *p* = .48				1.10, *p* = .29			

As can be seen in Table 4, it was not the case that male characters are depicted in higher status jobs compared to female characters. This association was significant only for Japan. However, when interpreting our findings, the very small numbers of depicted working roles need to be taken into account. Therefore, we also checked whether or not a female or male character was depicted in any working role at all. One could anticipate that male characters will be more likely to be shown in any working role compared to female characters. As Table 4 reveals, the association between male primary characters and the depiction of a working role was statistically significant for Austria, France, Japan, the Netherlands, Slovakia, and the United Kingdom. The association was not significant by conventional levels for Germany, Brazil, China, South Korea, Spain, Romania, and the United States.

**Table 4:** Status of working role by gender and by country and mere presence of a working role of the primary characters

Country	Status of working role				Mere presence of a working role			
	Men		Women		Men		Women	
	Low	High	Low	High	No	Yes	No	Yes
Austria								
*n* (%)	12 (57 %)	9 (43 %)	2 (33 %)	4 (67 %)	73 (78 %)	21 (22 %)	78 (93 %)	6 (7 %)
*χ* ^2^	1.06, *p* = .30				**7.96,** ***p*** **= .01**			
Brazil								
*n* (%)	8 (73 %)	3 (27 %)	1 (50 %)	1 (50 %)	37 (77 %)	11 (23 %)	24 (92 %)	2 (8 %)
*χ* ^2^	.41, *p* = .52				2.70, *p* = .10			
China								
*n* (%)	0 (0 %)	5 (100 %)	1 (33 %)	2 (67 %)	37 (88 %)	5 (12 %)	25 (89 %)	3 (11 %)
*χ* ^2^	1.91, *p* = .17				.02, *p* = .88			
France								
*n* (%)	8 (47 %)	9 (53 %)	1 (17 %)	5 (83 %)	34 (67 %)	17 (33 %)	53 (90 %)	6 (10 %)
*χ* ^2^	1.72, *p* = .19				**8.88,** ***p*** **= .003**			
Germany								
*n* (%)	4 (57 %)	3 (43 %)	1 (33 %)	2 (67 %)	40 (85 %)	7 (15 %)	62 (95 %)	3 (5 %)
*χ* ^2^	.48, *p* = .49				3.54, *p* = .06			
Japan								
*n* (%)	0 (0 %)	18 (100 %)	3 (27 %)	8 (73 %)	35 (66 %)	18 (34 %)	53 (83 %)	11 (17 %)
*χ* ^2^	**5.48,** ***p*** **= .02**				**4.38,** ***p*** **= .04**			
Netherlands								
*n* (%)	11 (46 %)	13 (54 %)	8 (80 %)	2 (20 %)	33 (58 %)	24 (42 %)	42 (81 %)	10 (19 %)
*χ* ^2^	3.34, *p* = .07				**6.63,** ***p*** **= .01**			
Romania								
*n* (%)	6 (75 %)	2 (25 %)	1 (25 %)	3 (75 %)	27 (77 %)	8 (23 %)	39 (91 %)	4 (9 %)
*χ* ^2^	2.74, *p* = .10				2.72, *p* = .10			
Slovakia								
*n* (%)	7 (44 %)	9 (56 %)	0 (0 %)	3 (100 %)	25 (61 %)	16 (39 %)	43 (94 %)	3 (7 %)
*χ* ^2^	2.08, *p* = .15				**13.42,** ***p*** **< .001**			
South Korea								
*n* (%)	7 (50 %)	7 (50 %)	4 (67 %)	2 (33 %)	52 (79 %)	14 (21 %)	32 (84 %)	6 (16 %)
*χ* ^2^	.47, *p* = .49				.46, *p* = .50			
Spain								
*n* (%)	4 (44 %)	5 (56 %)	2 (40 %)	3 (60 %)	17 (75 %)	9 (25 %)	45 (90 %)	5 (10 %)
*χ* ^2^	.03, *p* = .87				3.46, *p* = .06			
UK								
*n* (%)	8 (73 %)	3 (27 %)	3 (75 %)	1 (25 %)	39 (78 %)	11 (22 %)	48 (92 %)	4 (8 %)
*χ* ^2^	.01, *p* = .93				**4.16,** ***p*** **= .04**			
USA								
*n* (%)	9 (53 %)	8 (47 %)	6 (46 %)	7 (54 %)	43 (72 %)	17 (28 %)	42 (76 %)	13 (24 %)
*χ* ^2^	.14, *p* = .71				.33, *p* = .57			

### Multilevel Analyses

So far, we have observed how gender is related to the depiction of primary characters, and we have looked at single countries. Although such an analysis is useful, we are unable to explain why an association is found in one country and not in another. Thus, the question we want to ask is whether variations in the association between gender and character depiction can be explained by cultural differences between countries. In order to answer this question, hierarchical linear models (i.e., multilevel analyses) are needed (Raudenbush and Bryk 2002). Multilevel models are warranted when cases are clustered within countries. The advantages of multilevel analysis are that we can explain the individual-level variation in the dependent variable while statistically controlling the variation across levels of analysis and that we try to predict the variation of regression slopes by including constructs at the country level. In particular, we will examine whether the country differences in the relation between gender and character depiction can be explained by the five indices introduced previously.

Because our outcome variables are binary, we ran a logistic hierarchical non-linear model with the Logit-link function using PQL estimation (distribution at level-1: Bernoulli) with the statistical package HLM 7. The level-1 model includes the gender of the primary character. The level-2 model includes the respective index (i.e., Hofstede’s index, GLOBE, GDI, GII, or GGGI). Because the level-2 variables are correlated, we ran a separate model for each index.

In the first step, we computed the variance components in order to examine whether there is a significant amount of variance between the classes. The upper-level variance is significantly different from zero for the age of the primary character (*χ*^*2*^ = 55.29, *p* < .001), the product category body and cleaning products (*χ*^*2*^ = 79.03, *p* < .001), the product category technical products and cars (*χ*^*2*^ = 27.97, *p* < .001), working role shown (*χ*^*2*^ = 30.79, *p* < .001), and the status of the working role (*χ*^*2*^ = 27.28, *p* < .001). There was no significant amount of variance for the depicted setting: home (*χ*^*2*^ = 16.15, *p* = .06) and for the depicted setting: work (*χ*^*2*^ = 14.43, *p* = .11).

The results of the multilevel model are presented in Table 5. Because we included the grand-mean-centered terms for the gender indices, the effects of gender on the outcome variables must be interpreted as the effect of gender at the average level of a gender index (e.g., the effect of gender on depicted age at the average level of Hofstede’s masculinity index). For most outcomes, the level-1 effect of gender was statistically significant, confirming hypotheses Hypothesis 2 (character age), Hypothesis 4a (products for women), Hypothesis 4b (products for men), Hypothesis 5a (home setting), and Hypothesis 5b (work setting). However, we could not confirm the assumption that female primary characters are more likely to be seen in lower status working roles compared to male primary characters, who were theorized to be seen in higher status working roles (Hypothesis 6). Yet, female primary characters were less likely to be depicted in any working role compared to their male counterparts.

**Table 5:** Multilevel model predicting stereotypical depictions in TV ads

Model	Age	Product category: body/cleaning	Product category: technics/cars	Depicted setting: home	Depicted setting: work	Depicted in working role	Status working role (high)
Variables	*B* (*SE*)	*B* (*SE*)	*B* (*SE*)	*B* (*SE*)	*B* (*SE*)	*B* (*SE*)	*B* (*SE*)
Level-1							
Gender (Female)	−.106 (.14)***	1.73 (.17)***	−1.18 (.31)**	.84 (.14)***	−1.23 (.21)***	−.93 (.167)***	−.17 (.38)
Level-2							
Hofstede	.02 (.01)*	.01 (.01)	−.01 (.01)	.00 (.01)	−.01 (.01)	−.001 (.001)	.02 (.01)
Globe	−.02 (.51)	.67 (.70)	−.24 (.49)	.26 (.33)	.65 (.35)	.64 (.40)	−53 (.94)
GDI	2.88 (7.51)	3.27 (10.49)	9.12 (6.12)	.54 (4.78)	5.97 (5.09)	6.41 (6.02)	−17.56 (13.77)
GII	5.28 (1.80)*	−6.12 (3.27)	1.01 (2.28)	−5.7 (1.60)	−.32 (1.80)	−1.00 (2.12)	1.82 (4.61)
GGGI	−4.56 (3.47)	5.59 (5.13)	2.49 (3.61)	.29 (2.46)	3–28 (2.72)	2.10 (3.13)	−12.16 (−1.88)
Cross-level interactions							
Gender x Hofstede	−.01 (.01)	−.01 (.01)	.03 (.01)	−.01 (.001)	.00 (.91)	.00 (.01)	−01 (.02)
Gender x Globe	−.15 (.40)	.04 (.60)	−1.09 (1.09)	−.62 (.45)	−.25 (.70)	−.61 (.54)	.41 (1.00)
Gender x GDI	−2.39 (5.60)	1.94 (8.33)	−26.04 (14.00)	−2.34 (6.66)	3.67 (9.43)	−.61 (.54)	14.69 (14.91)
Gender x GII	−.29 (1.85)	.26 (3.14)	6.91 (4.46)	−.01 (2.21)	4.58 (2.80)	4.5 (2.26)	−.60 (4.53)
Gender x GGGI	1.54 (2.93)	3.47 (4.60)	−13.05 (6.85)	−2.76 (3.38)	−1.83 (.16)	−4.68 (3.72)	8.81 (7.47)

Effect of gender calculated with the Hofstede model; *Hofstede* Hofstede’s Masculinity Index, *Globe* GLOBE’s Gender Egalitarianism Index (Society Practices), *GDI* Gender-related Development Index, *GII* Gender Inequality Index, *GGGI* Global Gender Gap Index

* *p* < .05. ** *p* < .01. *** *p* < .001

Beyond the effects of the level-1 predictor, the main focus of the multilevel model lies on the cross-level interactions between the gender of the primary character and the gender indices. This interaction tests whether the variations between countries in the effects of gender on the outcome variables can be traced back to variations in the five gender indices. In order to answer this question, we first looked at the random-coefficients model. For the dependent variable age of the primary character, no systematic differences in the regression slope between the countries were observed (*χ*^2^ = 9.50, *p* = .39). The same was true for the dependent variables of product category body and cleaning products (*χ*^2^ = 7.08, *p* > .50), setting: home (*χ*^2^ = 10.11, *p* = .34), setting: work (*χ*^2^ = 5.05, *p* > .50), presence of a working role (*χ*^2^ = 7.19, *p* > .50), and status of the working role (*χ*^2^ = 12.45, *p* = .19). However, for the category of technical products and cars, there was a statistically significant variation that could be explained by level-2 variables (*χ*^2^ = 25.23, *p* < .01; not shown in Table). Thus, for most outcomes, there were no differences in the regression slope that can be explained by a specific culture. As can be seen in Table 5, we found no substantial cross-level interactions for all five gender indices. That is, none of the five gender indices was able to explain why there was a stronger (or weaker) association between the gender of the primary character and the outcome variables in a given country. Put formally, an increase in a gender index did not lead to an increase in the association between gender and the outcome variables.

## Discussion

Our study is the largest known to date on gender-role portrayals in advertising, and it is the first known with sample equivalence, using comparable TV programs to illuminate the effect of culture on gender-role portrayals. In addition, it is the first study known to test the role of gender indices for gender-role portrayals in advertising using multiple gender indices as independent variables and multiple gender-role variables as dependent variables. We have found significant differences among the countries investigated. Although every country showed traditional gender-role portrayals for some variables, some countries showed non-traditional gender-role portrayals for several variables and thus seem to use a more gender-equal approach toward gender roles.

For example, in the United Kingdom, no significant age differences were found between the share of male and female primary characters as well as male and female voiceovers, and similar shares of men and women were shown at home. Similarly, in the United States, men were not stereotypically associated with car/electronic products, approximately the same number of men and women were shown both at home and at work, and men and women were portrayed in work roles about evenly. These findings align with those of several previous research studies showing that in the United Kingdom and several European countries, gender portrayals have improved in recent years (Furnham and Mak 1999). By contrast, advertisements in several countries, such as Germany, were identified as highly traditional in the use of gender roles for almost all investigated variables. Across all countries, some variables tend to indicate more traditional gender-role portrayals than others. For example, for more than half the countries, the variables of voiceover, age, toiletries/household products, and setting produced significant gender differences.

Although we were able to observe differences between countries, we found that these differences cannot be explained by cultural or gender indices. The effect of a specific culture in shaping advertising messages is, therefore, smaller than commonly thought. This finding stands in contrast to Eisend’s (2010, p. 436) meta-analytic results showing that “gender stereotyping in advertising depends on developments related to gender equality in society rather than the other way around.” There are many potential explanations for why our study comes to a different conclusion. First and foremost, it is important to stress that no study of which we are aware has modeled the influence of culture in a multilevel model. As should be apparent, when looking at Tables 1, 2, 3 and 4, one could easily pick two or three countries and explain the observed differences by different scores on gender indices. However, such an analysis strategy does by no means confirm that an increase in a gender inequality index leads to an increase in gender stereotyping across countries. For this, a multilevel model is necessary.

Second, it is possible that we have found no relationship between the cultural variables and stereotyping because gender portrayals in advertising are lagging several years behind actual developments in society (Eisend 2010; Kim and Lowry 2005). Unfortunately, this cannot be sufficiently tested with the present data because we would need to draw a sample of TV ads over time. Third, because gender stereotypes in advertising are decreasing over time (Eisend 2010), our findings may be different simply because our sample is the most recent one, reflecting a declining influence of culture. That is, although advertising and its gender-role portrayals may still vary across cultures, in some cases, they may become more universal due to global markets and networked publics (Paek et al. 2011). Fourth, our study used equivalent samples in all countries which is not possible in a meta-analysis such as Eisend’s (2010). Fifth and finally, gender stereotypes might not be measured sufficiently by gender indices, and it may be that another index should be considered as more appropriate for measuring stereotypes (Williams and Best 1990).

### Limitations and Future Research Directions

As with every research project, our project has several limitations. These include a rather small sample size in some countries, which was also the case in several previous studies (Furnham and Paltzer 2010). More importantly, our sample includes countries from Asia, Europe, and the Americas, but none from Africa or Oceania. Related to this point, we were not able to sample all countries that we would have liked because of a lack of access to the TV channels. Generally, the number of countries was very small for a multilevel model. Larger and more diverse samples are therefore needed in future research. In addition, our sample was drawn in May 2014. Thus, it is able to represent only this specific period; additionally, seasonal variations might be a concern in research on advertising. Furthermore, although we strived to choose comparable TV networks, this is a daunting task because various countries have different broadcasting systems. In addition, analyzing only one TV channel for each country, even the most dominant one, might not be fully representative of the pool of TV ads from each country. Finally, we suggest that future research studies should attempt to analyze gender-role portrayals in television advertising over time using longitudinal approaches.

### Practice Implications

Practitioners in the countries we analyzed are called to raise their awareness for gender stereotypes in television ads. Even when practitioners reside in countries with high gender equality, gender stereotypes still prevail in television advertisements. Obviously, current (self-) regulatory efforts do not seem to be successful in implementing an unbiased representation of men and women in television ads. We suggest that advertising councils as well as advertising professionals should work toward a clearly defined set of recommendations about how men and women should be presented. We also suggest that advertising educators should sensitize students about gender role depictions in commercials, how they are observed using scientific methods, and what effects they may have on women and men.

### Conclusion

Our study was able to show that there appears to be a global pattern of gender stereotyping still at work. This finding is significant for two reasons. First, it is well known that gender stereotypes in advertising can influence gender-role stereotypes in society, further perpetuating gender roles and gender inequality (MacKay and Covell 1997; Oppliger 2007). Second, our findings clearly suggest that gender stereotypes in TV advertising can be found around the world, independent of a given gender equality status in a particular country. It follows that more progressive countries do not necessarily depict women—in terms of gender equality—in more progressive ways in television advertising. We hope our research helps to spur a discussion among scholars, advertisers, and regulators on the global dominance of gender stereotyping in advertising.

## Appendix

**Table 6:** Variables coded in the study

Variable	Codes	Examples
Product category	Body products & cleaning 1 = yes 0 = no	Body Care/Toiletries/Cosmetics/Beauty Products: Listerine mouth wash, sanitary napkins, soaps, shampoo, toothpaste, lotion, creams, face cleansers, diapers, etc. Household Cleaning Products/Kitchenware: detergents, washing powders, pans, etc.
	Technical products & cars 1 = yes 0 = no	Home Entertainment: CDs, DVDs, TVs, cameras, videos, games Mobile Phones/Providers: mobile phone providers, Samsung phones, etc. Computer/Information/Communications (not including mobile phones): Internet providers, laptops, iPad, etc. Automotive/Vehicles/Transportation/Accessories: Hyundai, Kia, Toyota, automobile tires, oil, etc.
Voiceover	0 = None 2 = Female 1 = Male 3 = Both	
Primary character	0 = Male primary character 1 = Female primary character 99 = No primary character	
Age	0 = 18–34 years 1 = 35 and older	
Dominant setting work	1 = work 0 = not work	Workplace (inside or outside)
Dominant setting home	1 = home 0 = not home	Home (inside residential space)
Depicted in working role	1 = yes 0 = not shown in any working role	
Status of working role	1 = higher status 0 = lower status	High Status Workers: business people, lawyer, doctor, musician, professor, actor, etc. Lower Status Workers: farmers, firefighters, house keeper, electrician, secretary, super market worker, seller in a store, etc.
Interaction with children	1 = yes 0 = no	

The full codebook with all instructions is available from the authors upon request

**Table 7:** Gender indices by country

Country	Hofstede’s masculinity index^a^	GLOBE’s gender egalitarianism index (society practices)^b^	Gender-related development index (GDI)^c^	Gender inequality index (GII)^d^	Global gender gap index^e^
Austria	79	3.09	.935	.056	.7266
Brazil	49	3.31	n/a	.441	.6941
China	66	3.05	.939	.202	.6830
France	43	3.64	.989	.080	.7588
Germany	66	3.10	.962	.046	.7780
Japan	95	3.19	.951	.138	.6584
Netherlands	14	3.50	.968	.057	.7730
Romania	42	n/a	.973	.320	.6936
Slovakia	100	n/a	1.000	.164	.6806
South Korea	39	2.50	.940	.101	.6403
Spain	42	3.01	.985	.100	.7325
UK	66	3.67	.993	.193	.7383
USA	62	3.34	.995	.262	.7463

^a^ A higher score means that the culture is more masculine, i.e., has more gender differentiation (Source: http://geert-hofstede.com)

^b^ A higher score means that the culture is more gender egalitarian (Emrich et al. 2004)

^c^ A higher score means that the society is closer to gender parity (Source: http://hdr.undp.org/en/content/table-5-gender-related-development-index-gdi)

^d^ A higher score means greater gender inequality in a society (Source: http://hdr.undp.org/en/content/table-4-gender-inequality-index)

^e^ A higher score means a smaller gender gap in a culture (Source: http://reports.weforum.org/global-gender-gap-report-2014)
